# Demographical, hematological and serological risk factors for *Plasmodium falciparum* gametocyte carriage in a high stable transmission zone in Cameroon

**DOI:** 10.1371/journal.pone.0216133

**Published:** 2019-04-25

**Authors:** Estelle Essangui, Carole Else Eboumbou Moukoko, Niels Nguedia, Michele Tchokwansi, Umaru Banlanjo, Franklin Maloba, Balotin Fogang, Christiane Donkeu, Marie Biabi, Glwadys Cheteug, Sylvie Kemleu, Emmanuel Elanga-Ndille, Léopold Lehman, Lawrence Ayong

**Affiliations:** 1 Malaria Research Unit, Centre Pasteur Cameroon, Yaounde, Cameroon; 2 Faculty of Sciences, University of Douala, Douala, Cameroon; 3 Faculty of Medicine and Pharmaceutical Sciences, University of Douala, Douala, Cameroon; 4 Faculty of Sciences, University of Buea, Buea, Cameroon; 5 Faculty of Sciences, University of Yaounde, Yaounde, Cameroon; 6 Faculty of Health Sciences, University of Buea, Buea, Cameroon; 7 Centre for Research in Infectious Diseases, Yaounde, Cameroon; Instituto Rene Rachou, BRAZIL

## Abstract

Presence of mature gametocyte forms of malaria parasites in peripheral blood is a key requirement for malaria transmission. Yet, studies conducted in most malaria transmission zones report the absence of gametocyte in the majority of patients. We therefore sought to determine the risk factors of both all-stage and mature gametocyte carriage in an area with high stable transmission of *Plasmodium falciparum* in Cameroon. Gametocyte positivity was determined using three complementary methods: thick blood smear microscopy, RT-PCR and RT-LAMP, whereas exposure to the infection was assessed by enzyme-linked immunosorbent assay. Of 361 malaria endemic residents randomly included in the study (mean age: 28±23 years, age range: 2–100 years, male/female sex ratio: 1.1), 87.8% were diagnosed with *P*. *falciparum* infection, of whom 45.7% presented with fever (axillary body temperature ≥37.5°C). Mature gametocyte positivity was 1.9% by thick blood smear microscopy and 8.9% by RT-PCR targeting the mature gametocyte transcript, *Pfs25*. The gametocyte positivity rate was 24.1% and 36.3% by RT-PCR or RT-LAMP, respectively, when targeting the sexual stage marker, *Pfs16*. Multivariate analyses revealed anemia as a common independent risk factor for both mature and all-stage gametocyte carriage, whereas fever and low anti-gametocyte antibody levels were independently associated with all-stage gametocyte carriage only. Taken together, the data suggest important differences in risk factors of gametocyte carriage depending on stage analyzed, with anemia, fever and low antiplasmodial plasma antibody levels representing the major contributing risk factors.

## Introduction

Despite considerable global efforts against malaria, the disease remains a major public health problem globally. In 2017, approximately 219 million malaria cases and 435,000 related deaths were recorded worldwide; the majority (92%) of which occurred in sub-Saharan Africa. In the same period, 390,130 of the cases were reported in Cameroon, where malaria remains highly endemic [1]. Malaria is caused by *Plasmodium* parasites, and 6 species (*Plasmodium falciparum*, *Plasmodium malaria*, *Plasmodium ovale*, *Plasmodium vivax*, *Plasmodium knowlesi and Plasmodium cynomolgi*) are responsible for malaria in humans [2,3]. *Plasmodium* parasites are transmitted through the bite of infected female *Anopheles* mosquitoes. Only carriers of the sexual parasite stages known as mature gametocytes are infectious to mosquitoes, and gametocyte carriage is dependent on host and parasite factors that may vary between individuals or geo-epidemiological transmission zones[4–6]. Gametocyte production in the human host is thought to initiate immediately after asexual division, resulting in the production and release of sexually committed rings that then develop into transmissible mature gametocyte also known as stage V gametocytes [7,8]. Indeed, only a small proportion (<5%) of the asexually multiplying parasite population often commit to sexual development, and only a small portion of the sexually committed parasites may develop into transmissible mature gametocyte forms [6]. The host and parasite factors that favor sexual commitment and maturation in malaria parasites are not fully understood but are believed to involve both parasite genetics and host immunological and/or stress-related responses [9]. Risk factors for mature gametocyte carriage in *P*. *falciparum* infected patients include patient age [10–12], gender [13], asexual parasite densities, [13–15], blood hemoglobin levels [15–17], infection duration [13], presence of a fever [5,11,14,16,18,19], as well as patient’s blood group [11,15]. Blood levels of *Plasmodium* gametocytes may also depend on the gametocytogenic potential of the infecting clones and the ability of the host environment to either promote or block gametocyte production *in vivo* [6,20]. Indeed, under certain stress conditions including antimalarial medication [21–24], anemia [15], and host immune activity [9], a strong gametocyte surge can be observed [25,26]. However, it is not known if all or some of the above factors constitute risk factors for sexual commitment and circulation of early gametocyte forms in infected persons. Understanding gametocyte production and dynamics as well as the associated risk factors from population-based studies is essential to effectively combating the disease in all transmission zones. Unfortunately, epidemiological studies on gametocyte carriage have been limited by lack of diagnostic tools capable of detecting all circulating gametocyte forms. With the advent of modern genomics technology, several molecular techniques including loop-mediated isothermal amplification and polymerase chain reaction based methods now exist for effective measurement of gametocyte carriage at endemic country levels. Amongst these, methods based on the early gametocytogenesis marker *Pfs16* and the mature gametocyte stage marker *Pfs25* are the most widely used [27–31].

In this study, both LAMP and PCR were used for high sensitivity detection of *P*. *falciparum* infection and gametocyte carriage, targeting the high abundance *PfExp1* transcript as asexual stage marker *and the Pfs16* and *Pfs25* transcripts as all-stage or mature gametocyte markers, respectively. This allowed to separately assess the demographic, hematologic and immunological risk factors of all-stage and mature gametocyte carriage in a region with high stable transmission of *P*. *falciparum* in Cameroon. Taken together, the data suggest important differences in risk factors of gametocyte carriage that depend on the gametocyte stage investigated, with anemia, fever and low antiplasmodial plasma antibody levels representing major contributing factors.

## Materials and methods

### Study site and population

The study was conducted in 2016 from February to September in the Esse health district of the Mefou-and-Afamba Division, Centre Region of Cameroon with field sampling undertaken in late February to early March and a second in late August to early September. Esse is a rural community of approximately 166 200 inhabitants covering a surface area of about 3 358 km^2^, and characterized by high stable perennial transmission of *P*. *falciparum* parasites. However, the populations have limited access to the available health facilities. The climate is equatorial with two dry seasons (December to February and July to August) and two rainy seasons (March to June and September to November), and the relief is consisted of plains and hills.

### Study design

This study was a cross-sectional study involving both children from two years of age and adults resident in two neighboring villages (Ongandi and Ngondibeles) in the Esse district in Cameroon. A non-probability sampling technique was adopted to recruit the maximum number of occupants of each household visited. Community sections with highest concentration of houses were targeted and the maximum number of consenting subjects in each household were enrolled for the study. Pregnant women were excluded from the study as they received intermittent preventive treatment comprising sulfadoxine and pyrimethamine known to increase gametocyte production in treated patients [21].The study was approved by the Cameroon National Ethics Committee (N°2014/05/458/L/CNERSH/SP), and administrative authorization was obtained from the Cameroon Ministry of Public Health. Written informed consent and assent forms were obtained from participants or their parents/tutors prior to inclusion in the study. Participation was voluntary, anonymous and without compensation. Structured questionnaires were used in obtaining clinical history (axillary body temperature, fever history, other known medical conditions, drug administration) as well as socio-demographic information (age, sex, place of residence including travel history). The presence of *P*. *falciparum* and other species of malaria parasites was detected by RDT testing (SD Bioline Malaria Ag *P*.*f*./Pan). Blood hemoglobin levels were determined using an automated hemoglobinometer (Hemoglobinometer Mission Hb, USA). All RDT positive persons were managed by a stand-by medical team and antimalarial treatments provided in accordance with national recommendations. *P*. *falciparum* parasitaemia was determined by microscopic examination of Giemsa-stained thick and thin blood smears. Parasite density was determined on the basis of the number of parasites per 200 leukocytes on a thick film, assuming total leukocyte counts of 8,000 cells/μL of whole blood.

### Blood sampling and RNA isolation

Venous blood samples (3 ml) were collected into EDTA vacutainer tubes and used for total RNA isolation as previously described [32,33]. Briefly, freed parasites were obtained from 2 ml of whole blood following saponin lysis. Total RNA was then extracted from each parasite pellet using 500 μl of TRI reagent, and re-solubilized in 30 μl of DEPC-treated water. The extract were immediately stored at -80°C until used.

### RT-LAMP and RT-PCR assays

*P*. *falciparum* gametocyte carriage was investigated by amplifying specific segments of the high-abundant *P*. *falciparum sexual stage* specific transcript, *Pfs16* (PF3D7_0406200) or the mature gametocyte stage-specific transcript, *Pfs25* (PF3D7_1031000). *Pfs16* is expressed in all gametocyte stages, including sexually committed schizonts and merozoites following induction [28]. Primers for RT-LAMP assays included primers F3/B3, FIP/BIP and LoopF/LoopB whereas primers for RT-PCR assay included primers F3 as forward primer and primer B3 as the reverse primer (Table 1).

**Table 1:** RT-LAMP and RT-PCR primer sequences.

Tests	Target gene	Primer name	Sequence (5’ to 3’)
RT-LAMP	*Pfs16*	F3	CTTCGCTTTTGCAAACCTG
		B3	CTAGCTGAGTTTCTAAAGGCAT
		FIP (F1c-F2)	GGTTTGCAAAGTTGAAGGGGATCAGATGCAAATGACAAAGCA
		BIP (B1c-B2)	CAGGAAGTTCTTCAGGTGCCTCACCTTGAGATAGTCCACCTT
		Loop F	CTTTTCCAGCGGGCTTTT
		Loop B	TCTTCATGCTGTTGGACCTAAT
RT-PCR	*Pfs16*	F3	CTTCGCTTTTGCAAACCTG
		B3	CATCTCCTTCGTCTCCTTCATC
	*Pfs25*	F3	AAGTTACCGTGGATACTGTATG
		B3	TGAGCATTTGGTTTCTCCAT

*Pfs16*: *Plasmodium falciparum* sexual stage 16 kD; *Pfs25*: *Plasmodium falciparum* sexual stage 25 kD

Primers for the *PfExp1*-based RT-LAMP (infection detection) as well as all RT-LAMP procedures were essentially as described previously [33]. Briefly, each assay was done in a total reaction volume of 25 μL, including 2.5μL of RNA extract, 2μL of primer mixes, 15μL of reconstituted enzyme (ISO-DR001, OptiGene), and 5.5μL of DEPC treated water (Diethyl pyrocarbonate). All LAMP assays were run in 16-microtube formats using the Genie II isothermal amplifier (OptiGene, UK), and at 65μC isothermal temperature for 1 hr. The specificity of each amplification was verified by including an annealing step of 98–70°C following which a single peak at around 87°C was expected.

RT-PCR was done using the AgPath-ID One-Step RT-PCR reagent kit designed for robust amplification of RNA targets in purified samples. Briefly, 2.5 μl RNA sample was added to a reaction mix comprising 0.4 μM of each forward or reverse primer, and 12.5 μl of RT-PCR buffer and 1 μl of RT-PCR enzyme. The following cycling conditions were adopted for both the *Pfs16* and *Pfs25* RT-PCRs: 45°C for 15 min, 95°C for 15 min, and 40 cycles of 95°C for 10 seconds, 50°C for 10 seconds, and 60°C for 1 min. All amplification products were verified by electrophoresis on 1.2% agarose gel stained with GelGreen II and visualized under ultraviolet light.

### Determination of anti-*Plasmodium falciparum* antibody responses

For each blood sample, plasma was collected and analyzed by direct ELISA to estimate total antibody levels against different antigen types, including a P. *falciparum* soluble protein extract and the sexual stage-specific recombinant proteins, Pfg27 (all gametocyte stages) and Pfs25 (mature gametocytes and ookinete stages). Total soluble antigens were extracted from *P*. *falciparum* strain 3D7 as previously described by freeze-thaw fractionation procedures [34,35]. Recombinant proteins corresponding to the gametocyte sexual stage antigens *Pfs25* and *Pfg27* were obtained from the BEI Resources (MRA-59 and MRA-1274, respectively) and used to determine host exposure to *Plasmodium* gametocytes.

For antibody ELISAs, 96-well microplates (F96 CERT-Maxisorp) were coated with each antigen (16 ng/well of antigen extract or 10 ng/well of recombinant proteins) in bicarbonate buffer (0.1 M, pH 8.5) by overnight incubation at 4°C. The plates were washed three times with 300 μL of wash buffer (PBS, pH 7.2) per well, and blocked for one hour at room temperature with 250 μl/well of 1% BSA in PBS. After washing three times with 300 μL/well of PBS buffer containing 0.05% Tween 20 (PBS/T), the plates were incubated with 100μL/well of diluted plasma (1:250 dilution in PBS/TB) for one hour at room temperature. The plates were then washed four times with 300 μL/well of wash buffer PBS/T. Human anti-IgG response was quantified using anti-human horseradish peroxidase (HRP)-conjugated IgG secondary Ab at 1:40 000 dilution (Thermo Fisher Scientific). Upon a 1 hour incubation at room temperature, the wells were washed four times with 300 μL/well of wash buffer PBS/T and subsequently incubated with tetramethylbenzidine (TMB) substrate solution for 10 minutes at room temperature. The developing color reaction was arrested by addition of 50 μl of 0.1 M sulphuric acid **(**H_2_SO_4_), and optical density (OD) was read at 450 nm. Plasma samples from European volunteer blood donors with no travel history to malaria endemic countries were used as negative controls. The OD was adjusted for background reactivity by including three blank wells in which wash buffer was used in place of plasma. The cut-off value was defined as a mean OD of negative control plus three times standard deviation (SD).

### Statistical analyses

Qualitative variables were expressed as frequencies while numerical variables were presented as mean +/- SD or 95% CI (95% confidence interval) if they were normally distributed. The univariate analysis was performed with either the exact Fisher test or the Chi-square test for the qualitative variables, and the Wilcoxon test or the ANOVA test for the quantitative variables. All statistical analyses were performed using Stata software (version 11 SE). The OR and association of each variable was estimated using a logistic regression model to account for the potential confounding factors. Only p <0.05 values were considered significant.

## Results

### Prevalence of *P*. *falciparum* infection and gametocyte carriage

To determine possible risk factors of gametocyte carriage in an area of high stable transmission of *P*. *falciparum*, a total of 361 participants with a mean age of 28 ± 23 (min-max: 2–100) years were randomly enrolled in this study. *P*. *falciparum* infection and gametocyte carriage were determined by light microscopy (asexual forms and mature gametocytes), by RT-PCR (all gametocyte forms and mature gametocytes), and by RT-LAMP (asexual and all gametocyte forms). Of the 361 participants, 56.8% were positive for *P*. *falciparum* infection by light microscopy (mean parasite density = 2, 041 parasites/ μl), 62.3% by RT-LAMP, and 62.1% by RDT. 317 (87.8%) were positive for *P*. *falciparum* infection in at least one of the three diagnostic methods used (S1 Table). 35.3% of these infected subjects were identified as submicroscopic infections (negative by microscopy but positive by RDT or molecular testing). Mature gametocyte carriage was detected in 7 (1.9%) participants when using light microscopy (mean gametocytaemia = 23 gametocytes/μl, range: 16–32 gametocytes /μl), and in 32 (8.9%) individuals when using RT-PCR (Table 2).

**Table 2:** Prevalence of *P*.*falciparum* infection and gametocyte carriage.

Tests	RDT	Microscopy		RT-LAMP		RT-PCR	
Target	All stages	Asexual stages	Mature gametocytes	*PfExp1* (asexual stages)	*Pfs16* (all sexual stages)	*Pfs16* (all sexual stages)	*Pfs25* (mature gametocytes)
Results	224 (62.1%)	205 (56.8%)	7 (1.9%)	225 (62.3%)	131 (36.3%)	87 (24.1%)	32 (8.9%)

Interestingly, all stage gametocytes (mature and sexually committed rings) were detected in up to 87 participants (24.1%) when using RT-PCR, and in up to 131 individuals (36.3%) when using RT-LAMP (Table 2). Positivity for either all stage or mature gametocyte carriage was significantly higher amongst the microscopically positive patients than the submicroscopically infected individuals (45.9% vs 28.6% for all stage, and 13.7% vs 2.7% for mature gametocyte carriage, p = 0.003 and 0.001, respectively). All microscopy-based gametocyte positives were also positive by *Pfs25* and *Pfs16*-based RT-PCR, and by *Pfs16*-based RT-LAMP. Furthermore, all *Pfs16* and *Pfs25*-based RT-PCR positives were also positive by *Pfs16*-based RT-LAMP, indicating that RT-LAMP was more sensitive at detecting all-stage gametocytes than RT-PCR. Concordance between the different gametocyte detection methods were 93.1% for light microscopy and *Pfs25*-based RT-PCR, 77.8% for light microscopy and *Pfs16*-based RT-PCR, and 65.7% for light microscopy and *Pfs16*-based RT-LAMP. Of the 131 gametocyte positives, 8 (6.1%) were negative for *P*. *falciparum* asexual stage infection by both light microscopy and *PfExp1*-based RT-LAMP. Taken together, the data indicate a high prevalence of gametocyte carriage in the study population, albeit only a small proportion (24.4%) of the all stage gametocyte positives were also positive for the transmissible mature stages.

### Demographic and clinical risk factors

**Fig 1:**
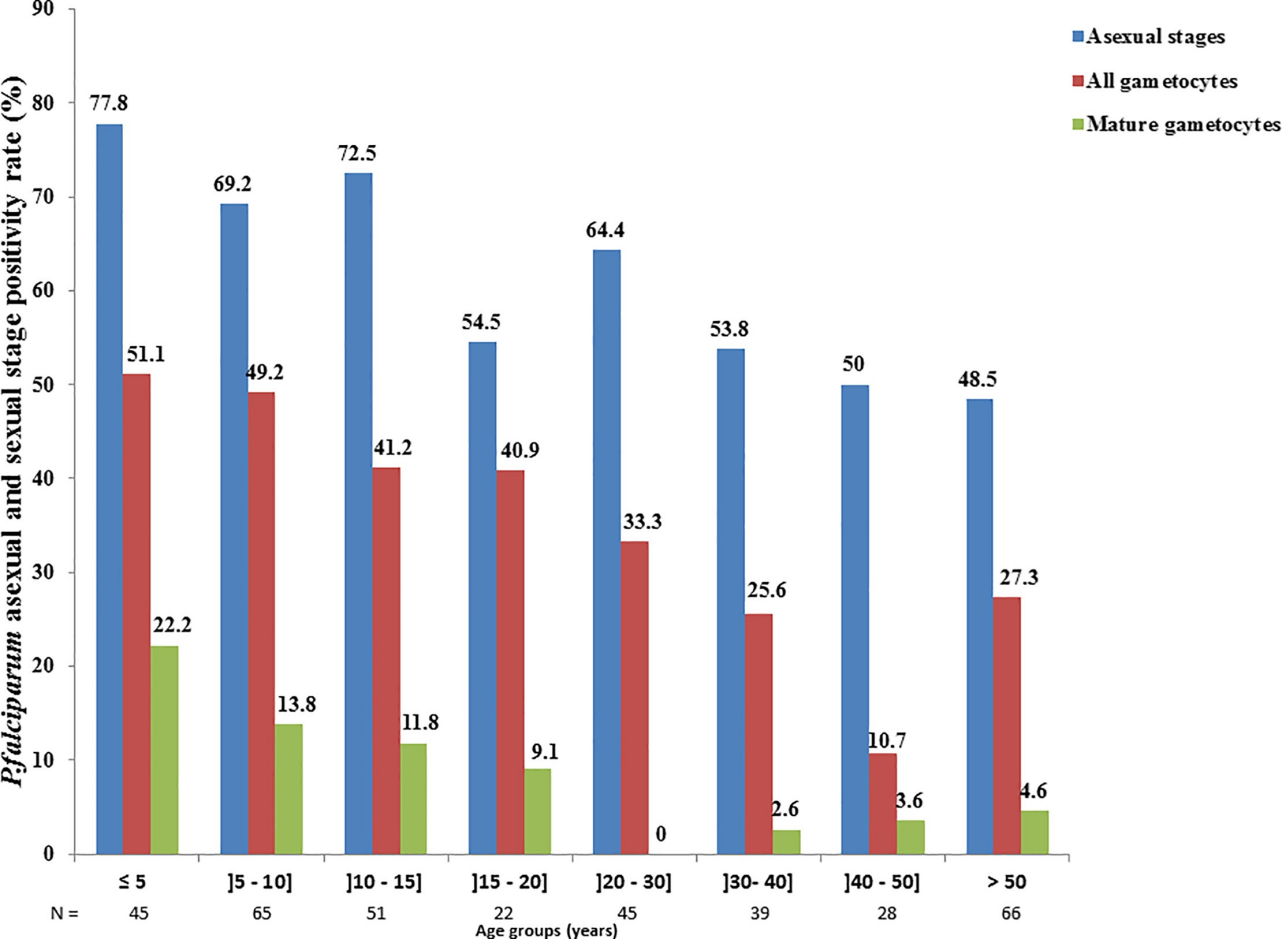
*P*. *falciparum* asexual and sexual stage positivity according to age. Data showing declining gametocyte positivity (all stage and mature gametocytes) with age. All stage gametocyte positivity was determined by RT-LAMP targeting the pan-gametocyte marker, *Pfs16*, whereas mature gametocyte carriers were identified by *Pfs25*-based RT-PCR. Similarly, positivity for P. falciparum asexual stages was determined by RT-LAMP targeting the asexual stage-specific marker, *PfExp1*.

To determine if age, gender, and clinical status (presence or not of a fever) constitute risk factors for gametocyte carriage, univariate analyses were performed based on the proportion of gametocyte carriage in each analytic group. As shown in Fig 1, gametocyte carriage decreased with age with the highest prevalence observed among children below 5 years of age. Univariate analyses (Table 3) also revealed young age (children ≤ 18 years) as a major risk factor for both all stage and mature gametocyte carriage (OR = 2.311, 95% CI: [1.490–3.584], p<10^−4^ and OR = 5.365, 95%CI: [2.151–13.381], p<10^−4^, respectively) No association was found between gametocyte carriage and gender. On the other hand, gametocyte carriage increased with the onset of clinical symptoms. Indeed individuals with axiliary temperature ≥37.5°C are twice as likely to carry gametocytes (all stages or mature gametocytes) compared to non-febrile individuals (OR = 2.161, 95%CI: [1.396–3.345], p = 0.001 and OR = 2.395, 95%CI: [1.133–5.063], p = 0.022, respectively) (Table 3).

**Table 3:** Univariate analyses of risk factors for all stage and mature gametocyte carriage.

	All sexual stages*		Mature gametocytes only**	
Variables	OR [95%CI]	P value	OR [95%CI]	P value
**Gender**				
Female, n = 174	1.390 [0.904–2.138]	0.134	1.917. [0.908–4.049]	0.088
Male, n = 187	1		1	
**Age group (years)**				
≤ 18	2.311 [1.490–3.584]	**<10**^**−4**^	5.365 [2.151–13.381]	**<10**^**−4**^
> 18	1		1	
**Fever (T≥37.5°C)**				
Yes n = 155	2.161 [1.396–3.345]	**0.001**	2.395 [1.133–5.063]	**0.022**
No n = 206	1		1	
**Anaemia**				
Yes n = 156	1.829 [1.185–2.824]	**0.006**	2.743 [1.281–5.877]	**0.009**
No n = 205	1		1	
**Parasitaemia (p/μl)**				
High ≥ 100 n = 149	1.070 [0.577–1.983]	0.831	3.562 [1.031–2.308]	0.045
Low < 100 n = 56	1		1	
**ABO Group**				
A, n = 93	1.059 [0.438–2.562]	0.831	2.310 [0.689–7.735]	0.175
B, n = 75	1.268 [0.516–3.117]	0.605	1.821 [0.541–6.127]	0.333
O, n = 164	1.015 [0.440–2.344]	0.972	3.024 [0.963–9.496]	0.058
AB, n = 28	1		1	
**Anti-*P*.*f* IgG**				
Low reactivity n = 181	2.371 [1.525–3.686]	**<10**^**−4**^	6.136 [2.307–16.326]	**<10**^**−4**^
High reactivity n = 180	1		1	
**Anti-*Pfs25* IgG**				
Low reactivity n = 180	1.314 [0.854–2.020]	0.214	1.325 [0.638–2.753]	0.450
High reactivity n = 181	1		1	
**Anti-*Pfg27* IgG**				
Low reactivity n = 180	2.039 [1.317–3.158]	**0.001**	2.381 [1.093–5.185]	**0.029**
High reactivity n = 181	1		1	

* Positivity to *Pfs16*

** Positivity to *Pfs25*

### Hematological and parasitological risk factors

As shown in Table 3, only anemia was associated with both all stage and mature gametocyte carriage in the study area (OR = 1.829, 95%CI: [1.185–2.824], p = 0.006). and OR = 2.743, 95%CI: [1.281–5.877], p = 0.009), respectively). No such significant associations were obtained between both all stage and mature gametocyte stage positivity and the ABO blood type (A, B, 0 and AB) or infection density (Table 3).

### Immunological risk factors

To investigate if gametocyte carriage also depended on host pre-exposure to the infection and/or gametocyte carriage, we determined the association between specific plasma antibody levels and positivity for either all stage or mature gametocyte carriage. All tested antigens were differentially recognized by the different plasma samples, with the highest reactivity achieved by using protein extracts (ELISA median OD: 1.368, range: 0.101–2.889). A median OD of 0.405 (range: 0.0807–2.1057) and 0.2762 (range: 0.0499–1.8104) was obtained when using the gametocyte-specific protein, *Pfg27* and the ookinete-specific surface protein *Pfs25*, respectively. As shown in Table 3, both all stage and mature gametocyte carriage were significantly associated with lower plasma antibody levels directed against the *P*. *falciparum* total antigen (OR = 2.371, 95%CI: [1.525–3.686], p<0.0001 and OR = 6.136, 95%CI: [2.307–16.326], p<0.0001). Positivity for all stage or mature gametocytes was also significantly associated with low antibody levels directed against the sexual stage-specific protein *Pfg27* (OR = 2.039, 95%CI: [1.317–3.158], p = 0.001, and OR = 2.381, 95%CI: [1.093–5.185], p = 0.029, respectively) but not against the mature gametocyte/ookinete stage-specific protein Pfs25 (OR = 1.314, 95%CI: [0.854–2.020], p = 0.214 and OR = 1.325, 95%CI: [0.638–2.753], p = 0.450, respectively). Taken together, the data suggest a strong association between host pre-exposure to the infection (asexual and sexual stages) and gametocyte positivity, with weakly immune individuals more likely to develop *P*. *falciparum* gametocytaemia.

### Independent risk factors of gametocyte carriage

To identify the demographic, hematological and serological risk factors that associated independently with gametocyte carriage in the population, multivariate analyses was done using young age, fever, anemia and low antibody responses against the P falciparum protein extract or sexual stage-specific *Pfg27* antigen. As shown in Table 4 below, anemia, fever and low antibody responses against the sexual stage-specific *Pfg27* protein were independently associated with all stage gametocyte positivity, whereas only anemia and low antibody responses against the *P*. *falciparum* protein extract were associated with mature gametocyte carriage. Together, the data suggest important differences between the risk factors for all stage and mature stage gametocyte carriage with anemia constituting the only common factor.

**Table 4:** Multivariate analysis of risk factors for all stage and mature gametocyte carriage.

	All sexual stages*		Mature gametocytes only**	
Variables	OR [95%CI]	P value	OR [95%CI]	P value
**Age (≤ 18 years)**	1.407 [0.8241–2.354]	0.193	2.529 [0.920–6.952]	0.072
**Anaemia**	1.675 [1.064–2.637]	**0.026**	2.362 [1.068–5.226]	**0.034**
**Fever (T°C≥37.5)**	1.738 [1.095–2.759]	**0.019**	1.473 [0.662–3.277]	0.343
**Low anti-*P*.*f* IgG**	1.612 [0.969–2.683]	0.066	3.352 [1.144–9.827]	**0.027**
**Low anti-*Pfg27* IgG**	1.644 [1.030–2.623]	**0.037**	1.532 [0.670–3.504]	0.311

* Positivity to *Pfs16*

** Positivity to *Pfs25*

## Discussion

This study aimed to determine the risk factors of gametocyte carriage in a high stable malaria transmission zone in Cameroon. We employed a recently developed RT-LAMP method [33] to include submicroscopically *P*. *falciparum* infected subjects in our analysis. Furthermore, we used a combination of three gametocyte detection techniques (RT-LAMP, RT-PCR, light microscopy) to identify active carriers of *P*. *falciparum* gametocytes in the study population. Exposure to gametocyte carriage was also investigated using an ELISA-based method. Together, our data show an extremely low prevalence (1.9%) of mature gametocyte carriage in the study area when using light microscopy. This finding is similar to those reported in other regions in Cameroon by Songue et *al* (0%), Van der Kolk et *al* (4.4%), and Sandeu et *al* (8.9%) using light microscopy as diagnostic method [36–38]. The mature gametocyte prevalence in our study population was only slightly increased to 8.9% when using RT-PCR as diagnostic method. We therefore sought to understand if such low prevalence rates also applied to total gametocytaemia that include both mature gametocytes and newly committed sexual stage parasites. Interestingly, the RT-PCR targeting the early gametocyte marker gene, *Pfs16*, revealed a gametocyte prevalence three-fold (24.1%) higher than the *Pfs25* RT-PCR. Considering that the *Pfs16* transcript is expressed at a two-fold higher level in mature gametocytes when compared to the *Pfs25* transcript [39], it is arguable that the observed difference in gametocyte prevalence may be due to differences in assay sensitivities. However, considering the generally low densities of mature gametocytes in the peripheral blood, it was conceivable that the increased sensitivity of the *Pfs16* target was due to the presence of early stage gametocytes (sexually committed rings) in the majority of infected blood samples. Indeed, *Plasmodium* gametocytogenesis is known to initiate in mid-trophozoite stage parasites leading to the release of sexually committed merozoites that infect and develop within circulating red blood cells as sexually committed rings before maturing through older gametocyte stages (stages I-V) [40]. However, only the sexually committed rings and mature stage V gametocytes appear in the circulation, as the remaining stages sequester in deep tissues, notably in the spleen and bone marrow [41,42]. Approximately 6% of the gametocyte positives (*Pfs16*-based RT-LAMP and *Pfs25*-based RT-PCR) were negative for *P*. *falciparum* infection as determined by light microscopy and *PfExp1*-based RT-LAMP. We interpreted such observations as corresponding to individuals who have recently cleared the infection either naturally or following antimalarial drug administration. This is consistent with several previous reports indicating that gametocyte positivity can proceed for several weeks after the clearance of asexual parasites, depending on the treatment or host immunity [43–45]

To determine possible risk factors of gametocyte carriage, we employed an RT-LAMP approach with previously demonstrated higher sensitivity when compared to RT-PCR [33] as main diagnostic method. Indeed, by RT-LAMP, we identified approximately 12% more gametocyte carriers compared to RT-PCR amplification of the same target gene. Additionally, all the RT-PCR gametocyte positives in our study were also positive by *Pfs16*-based RT-LAMP, justifying our use of RT-LAMP in the association studies. Consistent with findings elsewhere, young age, anemia [15–17] and fever [11,14,16,18,46,47] were identified as potential risk factors for both all stage and mature gametocyte carriage in the study area. In contrast to a previous study in Senegal [11] wherein various parasite density and the ABO blood groups (O and B) were identified as predictors of gametocyte positivity by light microscopy, we observed no such association with both all stage and mature gametocyte carriage based on molecular detection of the early gametocytogenesis *Pfs16* marker or the mature gametocyte marker *Pfs25* (cf Table 3). Interestingly, participants of age less than 18 were two times more likely to be positive for all stage gametocyte carriage, and five times more likely to be positive for the transmissible mature gametocyte stages than the adult participants. We therefore investigated the contribution of host immune factors such as anti-Plasmodial and anti-gametocyte antibody levels to gametocyte positivity using association analyses. Surprisingly, we observed a negative association between gametocyte carriage (all stage and mature gametocytes) and antibody levels against both the *P*. *falciparum* protein extract and the early gametocyte antigen, *Pfg27*. No such association was observed against antibody responses to the ookinete protein *Pfs25* (Table 3). Considering that a similar negative association has previously been identified between gametocyte positivity and antibody levels against another early gametocyte antigen *PfsEGXP* [48], the above findings suggest the involvement of anti-Plasmodial/anti-gametocyte directed antibodies in protection against gametocyte production and/or survival in pre-exposed individuals.

Of the identified risk factors, only anemia and low IgG antibody response against the *P*. *falciparum* extract were independently associated with mature gametocyte carriage, and only anemia, fever and low antibody responses to the Pfg27 protein associated independently with all stage gametocyte carriage. Indeed, the role of red blood cell volume (hematocrit) in *Plasmodium* gametocytogenesis has previously been demonstrated and thought to be related to the release of stress factors following massive red blood cell lyses that may induce *Plasmodium* gametocytogenesis [49]. Similarly, the association between gametocyte carriage and fever could result from a stress-dependent process involving host inflammatory responses favoring gametocytogenesis [19]. Meanwhile, our observation of a negative association between gametocyte carriage and the anti-plasmodial or anti-gametocyte IgG antibody responses is consistent with the occurrence of some level of anti-gametocyte immunity in the study population.

## Conclusion

In conclusion, gametocyte rates in the study population were comparatively high, and depended on test sensitivity and the gametocyte stage being targeted. On the basis of gametocyte positivity in the study population, we identified anemia as a common risk factor for both all stage and mature stage gametocyte carriage. Importantly, we identified low anti-Plasmodial antibody responses as major contributing factors to mature gametocyte carriage whereas low antibody responses to sexual stage antigens may associate strongly with gametocytogenesis and the initial release of early stage gametocytes into the peripheral blood. The observed negative association between the anti-gametocyte plasma antibody levels strongly support ongoing efforts to develop novel malaria transmission-blocking vaccines targeting *Plasmodium* gametocytogenesis.

## Supporting information

S1 Table Distribution of malaria positivity according to diagnostic method. (DOCX)
