# Supplementary material for: Demographical, hematological and serological risk factors for Plasmodium falciparum gametocyte carriage in a high stable transmission zone in Cameroon
Source: PLoS One. 2019 Apr 25;14(4):e0216133. doi: 10.1371/journal.pone.0216133 (PMC6483257; doi:10.1371/journal.pone.0216133)
Supplement: S1 Table — (DOCX) [file pone.0216133.s001.docx]

| Test outcome | Number of samples (% N*) |
| --- | --- |
| RDT only | 32 (8.9%) |
| Thick smear only | 23 (6.4%) |
| RT-LAMP *PfExp1* only | 36 (10%) |
| RDT + Thick smear | 37 (10.2%) |
| RDT + RT-LAMP *PfExp1* | 44 (12.2%) |
| Thick smear + RT-LAMP *PfExp1* | 34 (10%) |
| RDT+Thick smear+RT-LAMP *PfExp1* | 111 (30.7%) |
| Positivity in at least one test | 317 (87.8%) |
| ****N= 361*** |  |

**S1 Table. Distribution of malaria positivity according to diagnostic method**
